# Global and Ocular Hypothermic Preconditioning Protect the Rat Retina from Ischemic Damage

**DOI:** 10.1371/journal.pone.0061656

**Published:** 2013-04-23

**Authors:** Ezequiel M. Salido, Damián Dorfman, Melina Bordone, Mónica Chianelli, María Florencia González Fleitas, Ruth E. Rosenstein

**Affiliations:** Laboratory of Retinal Neurochemistry and Experimental Ophthalmology, Department of Human Biochemistry, School of Medicine, University of Buenos Aires/CEFyBO, CONICET, Buenos Aires, Argentina; Eye Hospital, Charité, Germany

## Abstract

Retinal ischemia could provoke blindness. At present, there is no effective treatment against retinal ischemic damage**.** Strong evidence supports that glutamate is implicated in retinal ischemic damage. We investigated whether a brief period of global or ocular hypothermia applied 24 h before ischemia (i.e. hypothermic preconditioning, HPC) protects the retina from ischemia/reperfusion damage, and the involvement of glutamate in the retinal protection induced by HPC. For this purpose, ischemia was induced by increasing intraocular pressure to 120 mm Hg for 40 min. One day before ischemia, animals were submitted to global or ocular hypothermia (33°C and 32°C for 20 min, respectively) and fourteen days after ischemia, animals were subjected to electroretinography and histological analysis. Global or ocular HPC afforded significant functional (electroretinographic) protection in eyes exposed to ischemia/reperfusion injury. A marked alteration of the retinal structure and a decrease in retinal ganglion cell number were observed in ischemic retinas, whereas global or ocular HPC significantly preserved retinal structure and ganglion cell count. Three days after ischemia, a significant decrease in retinal glutamate uptake and glutamine synthetase activity was observed, whereas ocular HPC prevented the effect of ischemia on these parameters. The intravitreal injection of supraphysiological levels of glutamate induced alterations in retinal function and histology which were significantly prevented by ocular HPC. These results support that global or ocular HPC significantly protected retinal function and histology from ischemia/reperfusion injury, probably through a glutamate-dependent mechanism.

## Introduction

Retinal ischemia plays a key role in some of the most common blinding diseases, such as diabetic retinopathy, glaucoma, retinal vascular occlusions, and retinopathy of prematurity, among others. In ischemic retinas, the metabolic requirements exceed the capacity of the circulation, and the tissue suffers from lack of nutrients, hypoxia, and build-up of waste products, which may ultimately lead to cell death [1]. In addition, reperfusion with oxygenated blood after ischemia has also the potential to aggravate ischemic damage, an effect known as reperfusion injury.

Glutamate, the main excitatory neurotransmitter in the retina, is toxic when present in excessive amounts. Therefore, an appropriate clearance of synaptic glutamate is required for the normal function of retinal excitatory synapses and neurotoxicity prevention. In fact, several lines of evidence strongly support the involvement of glutamate in retinal ischemic damage. In that context, it was shown that electrophysiological and neuronal damage following ischemia resembles that caused by exposure to glutamate [2]-[4], and that retinal ischemia induces a significant increase in glutamate release [5], [6]. Moreover, glutamate antagonists or inhibitors of glutamate release prevent retinal ischemic damage [7], [8]. At present, there is no effective treatment to protect the retina from ischemia/reperfusion (I/R) damage. Therefore, the development of resources to protect the retina against ischemia is a goal of vast clinical importance. An option to increase the retina’s resistance to ischemic injury is ischemic preconditioning (IPC), first introduced by Murry et al. [9]. The concept implies a brief subcritical ischemic challenge which could mobilize intrinsic protective mechanisms, increasing tolerance to a subsequent deleterious ischemia. The induction of ischemic tolerance has gained attention as a robust neuroprotective mechanism. In fact, Roth et al. [10] have shown that IPC affords the retina a greater degree of functional protection against ischemic damage than any known neuroprotective agent, making retinal IPC a particularly attractive area for further research. Although brief ischemia or hypoxia serve as prototypical IPC stimuli, ischemic tolerance can be induced by exposing animals or cells to diverse type of endogenous or exogenous stimuli that are not necessarily hypoxic or ischemic in nature, such as a moderate dose of bacterial lipopolysaccharide (LPS) [11] or hyperthermia [12], among others, supporting that one stressor can promote “cross-tolerance” to another [13]. However, one drawback of most preconditioning stimuli is that they are also capable of producing injury with only minor changes in their intensity or duration. In the case of LPS-induced retinal ischemic tolerance, for example, the intravitreal injection of 0.1 µg LPS is too weak to elicit protection, 1 µg LPS is highly effective as a preconditioning stimulus, but 5 µg LPS is injurious *per se* [11]. Similarly, in the case of ischemia-induced ischemic tolerance in the brain, 1 minute of ischemia is insufficient to elicit tolerance, whereas 2 to 5 minutes of ischemia elicit selective neuronal death [14], [15]. This lack of a suitable safety margin greatly limits the therapeutic utility of IPC in many clinical settings. It has been shown that transient hypothermia is an effective preconditioning stimulus for inducing tolerance to ischemic injury in several systems, such as myocardium and brain [16], [17]. An attractive feature of hypothermic preconditioning (HPC) is that it possesses a broad safety margin [18]. In that sense, it was shown that a moderate level of hypothermia does not represent a direct risk to neurons. Consequently, hypothermia can serve as an effective preconditioning stimulus without presenting a direct risk to neural tissue [18]. In fact, HPC is now considered to be one of the most potent cardioprotective strategies available and is used clinically to protect the heart against ischemia [19]. As for the retina, it was reported that local hypothermia during vitrectomy reduces retinal damage in pressure induced mild ischemia [20]. Faberowski et al. [21] demonstrated a significant neuroprotection of local hypothermia in a ligation-induced I/R model in the rat retina, and the protective effect of lowering body temperature during retinal ischemia has also been observed in high intraocular pressure models in rabbit and mouse [22], [23]. In addition, lowering body temperature to 33°C during ischemia and reperfusion in the retina markedly reduces ischemia-induced cell loss in the ganglion cell layer (GCL) [24]. Although these results indicate that intraischemic hypothermia limits retinal ischemic damage, the efficacy of HPC against retinal I/R injury was not previously examined. Thus, the aim of the present study was to analyze the capacity of a short period of global or ocular hypothermia applied before the ischemic event, for protecting the retinal function and histology against I/R damage.

## Materials and Methods

### Ethics Statement

All animal procedures were in strict accordance with the ARVO Statement for the Use of Animals in Ophthalmic and Vision Research. The ethic committee of the School of Medicine, University of Buenos Aires (Institutional Committee for the Care and Use of Laboratory Animals, (CICUAL)) approved this study.

### Animals

Male *Wistar* rats (average weight 250±40 g) were housed in a standard animal room with food and water *ad libitum* under controlled conditions of humidity, temperature (21±2°C), and luminosity (200 lux), under a 12-hour light/12-hour dark lighting schedule (lights on at 7∶00 AM). A total number of 104 animals were used for the experiments, distributed as follows: for the study of the effect of ocular cooling on body temperature: 4 animals; for the studies of global hypothermia: 20 animals submitted to ischemia in one eye and a sham procedure in the contralateral eye which were maintained in normothermic conditions (10 animals) or submitted to global hypothermia 24 h before ischemia (10 animals); for the studies of ocular hypothermia: 20 animals submitted to ischemia in one eye and a sham procedure in the contralateral eye which were maintained in normothermic conditions or were submitted to ocular hypothermia 24 h before ischemia; for the studies of the effect of ocular hypothermia on glutamate uptake and glutamine synthetase: 40 animals with ischemia in one eye and a sham procedure in the contralateral eye with or without ocular hypothermia, and for the studies of the effect of ocular hypothermia on glutamate injury: 20 animals injected with vehicle in one eye and glutamate in the contralateral eye, with or without ocular hypothermia applied 24 h before injections.

### Ischemia Methodology

Animals were anesthetized with ketamine hydrochloride (150 mg/kg) and xylazine hydrochloride (2 mg/kg) administered intraperitoneally. After topical instillation of proparacaine, the anterior chamber was cannulated with a 30-gauge needle connected to a pressurized bottle filled with sterile normal saline solution. Retinal ischemia was induced by increasing intraocular pressure (IOP) to 120 mmHg for exactly 40 min, as previously described [2]. With this maneuver, complete ocular ischemia was produced, characterized by loss of electroretinogram (ERG) b-wave and cessation of flow in retinal vessels, determined by funduscopic examination. The contralateral eye was cannulated without raising IOP. During and after (before returning rats to the animal house) ischemia, animals were kept normothermic with heated blankets. A few animals (less than 5%) in which cataracts developed due to lens injury, were not used any further in the experiments.

### Global and Ocular Hypothermia

Global hypothermia was achieved by applying ice packs to the animal body while they were in a state of anesthesia induced by ketamine and xylazine. The duration and depth of hypothermia was fixed at 20 minutes and 33°C, as described by Yunoki et al. [18]. The total duration of hypothermia was actually longer than 20 min, because approximately 10 min were required to cool the animals from 37.5°C, and an additional 10-min period was required to rewarm the animals by thermal covers. When the body temperature was lowered to 33°C, the ocular temperature was 32°C. Control animals were anesthetized for the same period without cooling. For core temperature measurements, a rectal probe was inserted 6 cm into the rectum. In case the body temperature of a rat deviated more than 0.5°C from the set point for more than 5 min, this rat was excluded from the study.

**Figure 1:**
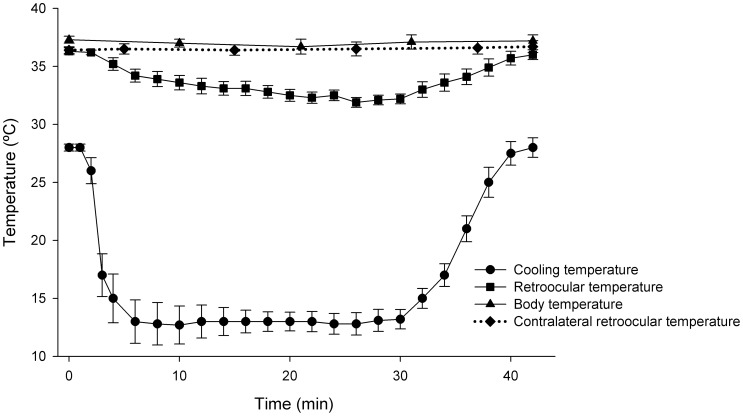
Temporal course of body, ipsilateral, and contralateral retroocular temperature during unilateral ocular cooling. A flow of ultrasound transmission gel cooled at 13°C was applied to one eye, which achieved a temperature of 32°C. Note that when one eye was perfused with the cooling gel, the contralateral eye, and the body temperature remained normothermic. Data are mean ± SE (n: 4 animals).

For ocular cooling, a flow of standard water-soluble ultrasound transmission gel (Gel Ultra Son, Dafton Acoplantes, Buenos Aires, Argentina) cooled at 13°C was applied to one eye while limiting systemic hypothermia. In a pilot experiment, the relationship between ocular and systemic temperatures was determined by simultaneously monitoring retroocular and rectal temperatures. In these experiments, an incision was made 2 cm lateral to the eye and a thermistor needle was implanted behind the ocular globe. The results of this study showed that when the gel temperature was lowered to 13°C, and the rectal temperature remained at 37.5°C, the ocular globe temperature achieved was 32°C, while the temperature of the contralateral eye remained normothermic (Figure 1). These parameters for rectal and ocular temperatures were used in subsequent experiments in which the placement of the thermistor behind the ocular globe was omitted because of the potential for direct injury by the probe. The duration of ocular hypothermia was fixed at 20 minutes with a targeted retroocular temperature of 32°C.

### Electroretinography

Electroretinographic activity was assessed as follows: after 6 h of dark adaptation, rats were anesthetized under dim red illumination. Phenylephrine hydrochloride and tropicamide were used to dilate the pupils, and the cornea was intermittently irrigated with balanced salt solution to maintain the baseline recording and to prevent keratopathy. Rats were placed facing the stimulus at a distance of 20 cm. All recordings were completed within 20 min and animals were kept warm during and after the procedure. A reference electrode was placed through the ear, a grounding electrode was attached to the tail, and a gold electrode was placed in contact with the central cornea. A 15 W red light was used to enable accurate electrode placement. This maneuver did not significantly affect dark adaptation and was switched off during the electrophysiological recordings. ERGs were recorded from both eyes simultaneously and ten responses to flashes of unattenuated white light (5 ms, 0.2 Hz) from a full-field/Ganzfeld stimulator (light-emitting diodes) set at maximum brightness (9 cd s/m^2^ without filter) were amplified, filtered (1.5-Hz low-pass filter, 1000 high-pass filter, notch activated) and averaged (Akonic BIO-PC, Buenos Aires, Argentina). The a-wave was measured as the difference in amplitude between the recording at onset and the trough of the negative deflection and the b-wave amplitude was measured from the trough of the a-wave to the peak of the b-wave. Electrophysiological responses were averaged for each run. Runs were repeated 3 times with 5 min-intervals to confirm consistency and the mean of these 3 runs was used for subsequent analysis. The mean peak latencies and peak-to-peak amplitudes of the responses from each group of rats were compared.

Oscillatory potentials (OPs) were assessed as previously described [2]. Briefly, the same photic stimulator with a 0.2 Hz frequency and filters of high (300 Hz) or low (100 Hz) frequency were used. The amplitudes of the OPs were estimated by measuring the heights from the baseline drawn between the troughs of successive wavelets to their peaks. The sum of three OPs was used for statistical analysis.

### Histological Evaluation

Rats were sacrificed and their eyes were immediately enucleated, immersed for 24 h in a fixative containing 4% formaldehyde in 0.1 M phosphate buffer (pH 7.2) and embedded in paraffin. Eyes were sectioned (5 µm) along the vertical meridian through the optic nerve head. Microscopic images were digitally captured with a Nikon Eclipse E400 microscope (illumination: 6-V halogen lamp, 20 W, equipped with a stabilized light source) via a Nikon Coolpix s10 camera. Sections were stained with hematoxylin and eosin (H&E) and analyzed by masked observers. The total retinal, photoreceptor outer segment (PS), outer nuclear layer (ONL) outer plexiform layer (OPL), inner plexiform layer (IPL), and inner nuclear layer (INL) thickness (in µm) was measured for each eye. Measurements (400x) were obtained at 1 mm dorsal and ventral from the optic disc. For each eye, results obtained from four separate sections were averaged and the mean of 5 eyes was recorded as the representative value for each group.

### Immunohistochemical Study

Antigen retrieval was performed by heating (90°C) slices for 30 min in citrate buffer and then preincubated with 2% normal horse serum, 0.1% bovine serum albumin, and 0.4% Triton X-100 in 0.01 M phosphate-buffered saline for 1 h. The sections were then incubated overnight at 4°C with a mouse monoclonal anti-Brn3a antibody (1∶500 Millipore, Temecula, CA, USA). An anti- anti-goat secondary antibody conjugated to Alexa Fluor 568 (1∶500; Molecular Probes, Grand Island, NY, USA) was used. After immunostaining, the sections were mounted with antifade medium with the fluorescent nuclear stain DAPI (Vector Laboratories, Burlingame, CA, USA). Some sections were treated without the primary antibodies to confirm specificity. An Olympus BX50 microscope (Olympus, Tokyo, Japan) was used for microscopic observations. Comparative digital images from different samples were grabbed using identical time exposition, brightness, and contrast settings.

### L-^3^H-glutamate Uptake Assessment

The influx of L-^3^H-glutamate was assessed in a crude synaptosomal fraction of rat retinas, as previously described [2]. Retinas were homogenized (1∶9 w/v) in 0.32 M sucrose containing 1 mM MgCl_2_, and centrifuged at 900 *g* for 10 min at 4°C. Nuclei-free homogenates were further centrifuged at 30,000 *g* for 20 min. The pellet was immediately resuspended in buffer HEPES-Tris, containing 140 mM NaCl, 5 mM KCl, 2.5 mM CaCl_2_, 1 mM MgCl_2_, 10 mM HEPES, 10 mM glucose, (adjusted to pH 7.4 with Tris base) and aliquots (100 - 300 µg protein/100 µl) were incubated with 100 µl of L-^3^H-glutamate (500,000–800,000 dpm/tube, specific activity 17.25 Ci/mmol) and 10 µM glutamate. After 5 min, glutamate uptake was terminated by adding 4 ml of ice cold HEPES-Tris buffer. The mixture was immediately poured onto Whatman GF/B filters under vacuum. The filters were washed twice with 4 ml-aliquots of ice-cold buffer and the radioactivity on the filters was counted in a liquid scintillation counter. Non-specific uptake of L-^3^H-glutamate into synaptosomes was assessed by adding an excess of glutamate (10 mM).

### Glutamine Synthetase Assessment

Each retina was homogenized in 200 µl of 10 mM potassium phosphate, pH 7.2. Glutamine synthetase activity was assessed as described [2]. Reaction mixtures contained 150 µl of retinal homogenates and 150 µl of a stock solution (100 mM imidazole-HCl buffer, 40 mM MgCl_2_, 50 mM β-mercaptoethanol, 20 mM ATP, 100 mM glutamate and 200 mM hydroxylamine, adjusted to pH 7.2). Tubes were incubated for 15 min at 37°C. The reaction was stopped by adding 0.6 ml of ferric chloride reagent (0.37 M FeCl_3_, 0.67 M HCl, and 0.20 M trichloroacetic acid). Samples were placed for 5 min on ice. Precipitated proteins were removed by centrifugation, and the absorbance of the supernatants was read at 535 nm against a reagent blank. Under these conditions, 1µmol of γ-glutamylhydroxamic acids gives an absorbance of 0.340. Glutamine synthetase specific activity was expressed as µmoles of γ-glutamylhydroxamate per hour per milligram of protein.

### Intravitreal Injection of Glutamate

Animals were anesthetized as previously described. A drop of proparacaine (0.5%) was administered in each eye for local anesthesia. With a Hamilton syringe (Hamilton, Reno, NV, USA) and a 30-gauge needle, 4 µl of 0.3 M glutamate (estimated final concentration 20 mM, considering a vitreous volume of 60 µl [25]) in sterile pyrogen-free saline were injected into one eye of anesthetized rats, while an equal volume of vehicle (saline solution) was injected in the fellow eye. Injections were applied at 1 mm of the limbus and the needle was left in the eye for 60 seconds; this small volume prevented the increase in IOP and volume loss. The dose of glutamate was selected on the basis of a previous report [2]. Retinal function and histology were examined 7 days after intravitreal injections of vehicle or glutamate.

### Protein Level Assessment

Protein content was determined by the method of Lowry et al. [26], using bovine serum albumin as the standard.

### Statistical Analysis

Statistical analysis of results was made by a two-way analysis of variance (ANOVA) followed by a Tukey’s test, as stated. For all analyses, a value of *P*<0.05 was considered significant.

## Results

### Global Hypothermic Preconditioning

**Figure 2:**
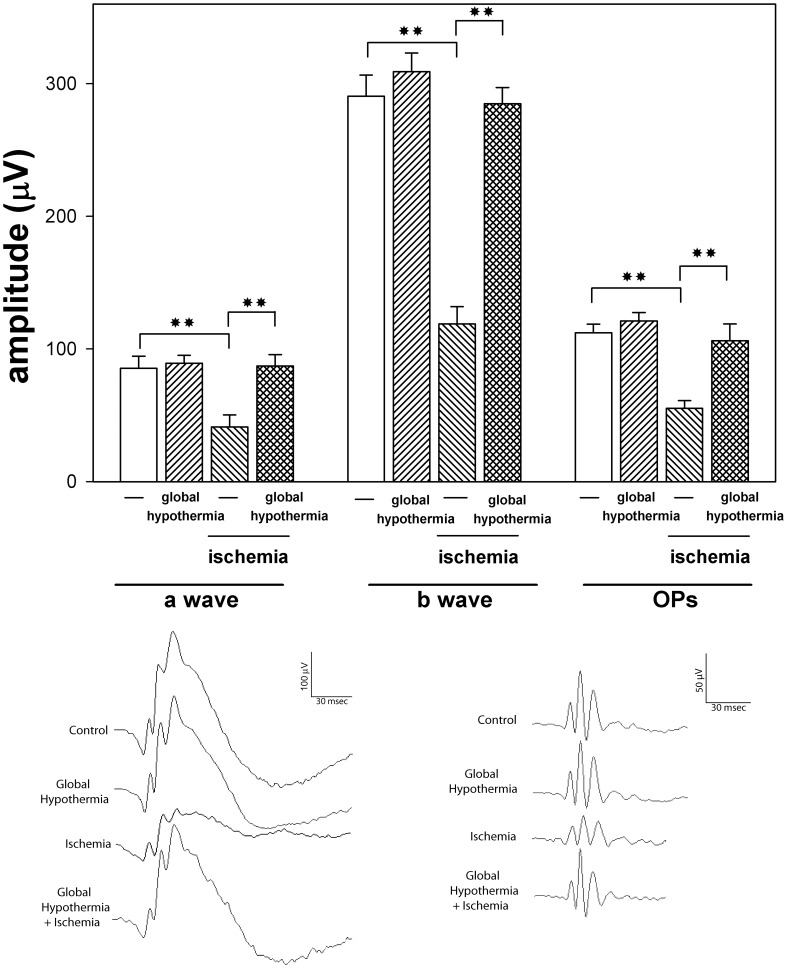
Effect of global HPC on retinal function. Scotopic ERGs from rats subjected to 40-min ischemia without or with global hypothermia applied 24 h before ischemia, and registered 14 days after ischemia. Upper panel: average amplitudes of scotopic ERG a-wave, b-wave, and OPs. A significant decrease in the ERG a-wave, b-wave, and OP amplitude was observed in eyes exposed to 40-min ischemia without global HPC. In animals submitted to global HPC, a significant preservation of the ERG a-wave, b-wave, and OP amplitude was observed. Data are mean ± SE (n: 10 eyes/group). **P<0.01, by Tukey’s test. Lower panel: Representative scotopic ERG and OP traces from a non-ischemic eye without or with local HPC, and from an eye subjected to 40-min ischemia without or with global HPC applied 24 h before ischemia.

Figure 2 depicts the effect of global HPC on retinal functional damage induced by increasing IOP to 120 mm Hg for 40 min. One day before ischemia, a group of animals were kept at 33°C for 20 min, whereas control animals were maintained in normothermic conditions. The average amplitudes of ERG a- and b- waves and OPs in non-ischemic eyes or 14 days after 40-min ischemia without or with global HPC, as well as representative scotopic ERG traces from rat eyes submitted to these treatments are shown in Figure 2. A significant decrease in ERG a- and b- wave amplitude was observed at 14 days after ischemia, while their latencies remained unchanged (data not shown). Global HPC significantly prevented the decrease in ERG a- and b-wave amplitude induced by I/R. A similar profile was observed for OPs, as shown in Figure 2. In non-ischemic eyes, global hypothermia did not affect the ERG.

**Figure 3:**
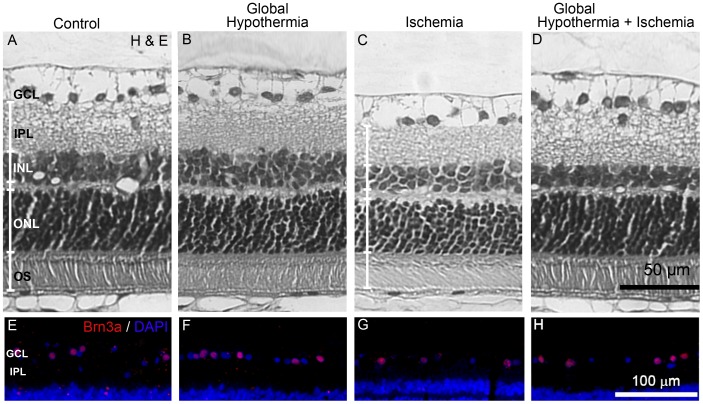
Effect of global HPC on retinal histology. Upper panel: Representative photomicrographs showing histological appearance of non-ischemic retinas without (A) or with global HPC (B), ischemic retinas 14 days after 40 min of ischemia without global HPC (C), and ischemic retinas from animals submitted to global HPC, 24 h before ischemia (D). Severe retinal damage is shown in the retina from eyes submitted to ischemia without global HPC, whereas in animals submitted to global HPC, the retinal structure was notably preserved. Lower panel: Immunohistochemical detection of Brn3a(+) cells in the GCL from all the experimental groups. A decrease in GCL Brn3(+) cell number was observed in ischemic retinas without global HPC (G) as compared with non-ischemic eyes (E), whereas global HPC, which showed no effect in control eyes (F), partly preserved Brn3a(+) cell count in ischemic eyes (H). GCL, ganglion cell layer; IPL, inner plexiform layer; INL, inner nuclear layer; ONL, outer nuclear layer, OS, outer segment of photoreceptors.

Figure 3 and Table 1 show the effect of a 20-min period of global hypothermia on retinal histology. Figure 3A shows a representative photomicrograph of non-ischemic retinas. Fourteen days after 40-min ischemia, typical histopathological features of ischemic damage were observed, showing marked reduction in the total retinal, outer nuclear layer (ONL), inner nuclear layer (INL), and inner plexiform layer (IPL) thickness (Figure 3C), and a significant decrease in the number of Brn3a(+) cells in the ganglion cell layer (GCL) (Figure 3G), as compared with control retinas (Figure 3A and 3E). Global HPC (Figure 3D) which did not show effect *per se* (Figure 3B and 3F), significantly prevented the decrease in total retinal, INL, IPL thickness, and in the number of Brn3a(+) cells in the GCL (Figure 3H).

**Table 1:** Histological analysis of the effect of global HPC on retinal ischemic damage.

	Total	PS	ONL	OPL	INL	IPL	Brn3a (+) cell number
Control	132.0±5.8	27.0±1.4	40.0±1.4	6.1±0.3	21.8±1.4	34.3**±**2.8	5.5**±**0.2
Hypothermia	134.2±4.3	27.1±1.2	39.9±1.5	6.2±0.4	20.2±0.8	35.0**±**0.8	5.6**±**0.2
Ischemia	99.7±2.0**	24.7±2.2	31.3±1.0*	6.0±0.9	15.3±0.5**	18.9**±** 1.1**	3.4**±** 0.3**
Global HPC+ischemia	116.0±4.3* ^,^ a	27.2±1.6	36.2±3.0	5.9±1.5	17.5±0.7* ^,^ b	30.6**±** 1.0 a	4.9**±**0.4* ^,^ a

Total retinal and retinal layer thicknesses (in µm) in non-ischemic or ischemic eyes without or with global HPC. Retinal ischemia induced a significant decrease in total retinal, ONL, INL and IPL thickness, and in GCL Brn3a(+) cell number. The alterations in total retinal, INL, and IPL (but not ONL) thickness, and in retinal ganglion cell number were partially prevented by global HPC, which showed no effects in non-ischemic eyes on these parameters. Data are mean ± SEM (n = 5 eyes per group).

* *P*<0.05 and,

** *P*<0.01 vs non-ischemic eyes;

a p<0.05 and,

b p<0.01 vs. ischemic eyes without global HPC, by Tukey’s test.

PS, photoreceptor outer and inner segment; OPL, outer plexiform layer; ONL, outer nuclear layer; IPL, inner plexiform layer; INL, inner nuclear layer.

### Ocular Hypothermic Preconditioning

**Figure 4:**
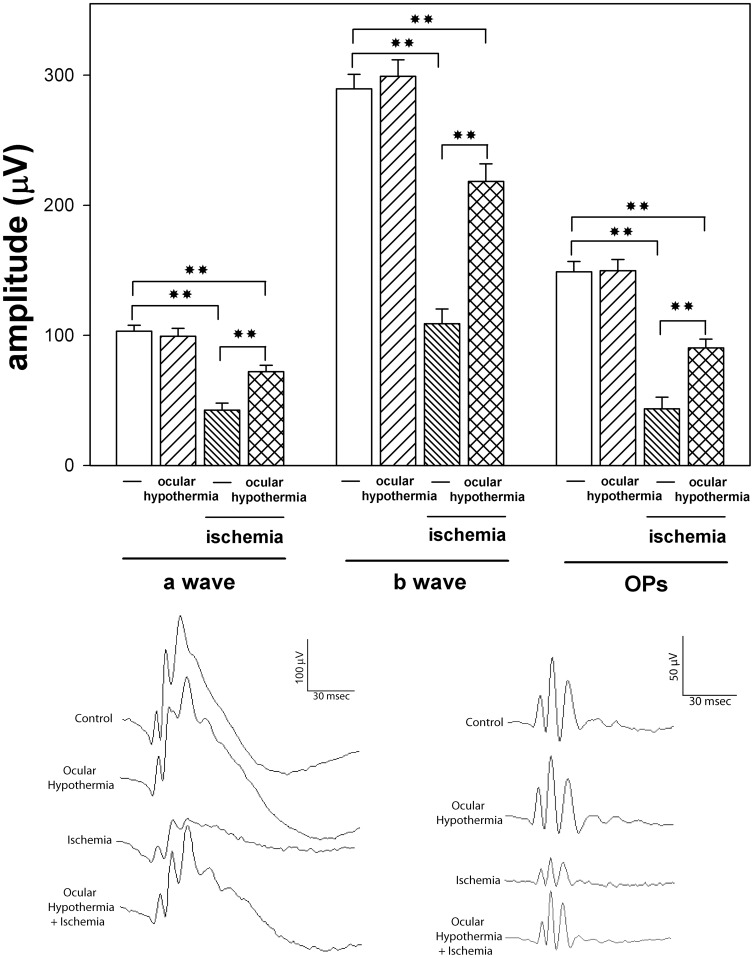
Functional protection against I/R damage induced by ocular HPC . Upper panel: Average amplitudes of scotopic ERG a-wave, b-wave and OPs. In eyes submitted to 40-min ischemia, local HPC significantly prevented the decrease in the amplitude of ERG b- (but not a-) wave and OP amplitude assessed 14 days after 40-min ischemia. Data are mean ± SE (n: 10 eyes/group). **P<0.01, by Tukey’s test. Lower panel: Representative scotopic ERG and OP traces from all the experimental groups.

**Figure 5:**
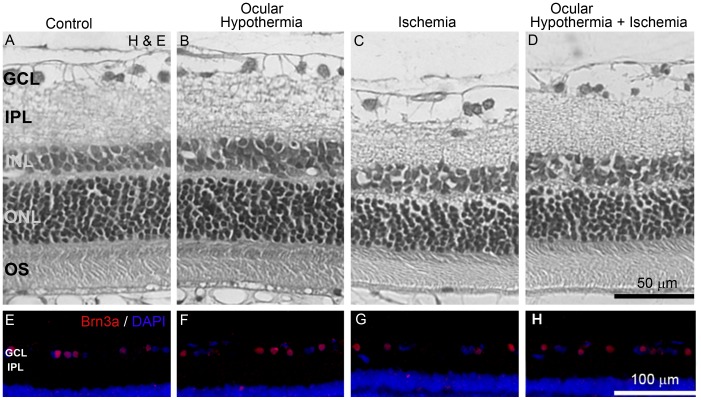
Histological protection against I/R damage induced by local HPC. Upper panel: Severe retinal damage is shown in the retina from eyes submitted to ischemia without local HPC (C) as compared with non-ischemic eyes (A), whereas local HPC applied 24 h before ischemia (D), notably preserved the retinal structure. Lower panel: A similar profile was observed for Brn3a(+) cell number in the GCL. Local HPC did not affect retinal histology and Brn3a(+) cell number in non-ischemic eyes (B and F, respectively). GCL, ganglion cell layer; IPL, inner plexiform layer; INL, inner nuclear layer; ONL, outer nuclear layer, OS, outer segment of photoreceptors.

The protective effect of ocular HPC against I/R injury was analyzed at functional and histological level. The average amplitudes of scotopic ERG a- and b- waves and OPs in control animals or animals submitted to ischemia, without or with ocular hypothermia applied 24 h before ischemia, as well as representative scotopic ERG traces from rats submitted to these treatments are shown in Figure 4. Ocular hypothermia significantly prevented the decrease in the ERG a-wave, b-wave, and OP amplitude induced by I/R. The protective effect of ocular hypothermia was also evident at histological level (Figure 5 and Table 2). Ocular hypothermia significantly prevented the decrease in total retinal, INL, and IPL thickness, and the number of Brn3a(+) cells in the GCL induced by 40- min ischemia. Ocular hypothermia did not affect the decrease in ONL thickness induced by I/R (Table 2).

**Table 2:** Histological analysis of the effect of ocular HPC on retinal ischemic damage.

	Total	PS	ONL	OPL	INL	IPL	Brn3a (+) cell number
Control	133.9±3.5	27.5±1.1	41.6±1.5	6.4±0.4	20.5±0.4	35.3**±**2.5	5.2**±**0.3
Hypothermia	135.7±3.9	26.7±0.9	40.9±1.0	6.6±0.3	19.5±1.0	34.8**±**1.0	5.6**±**0.2
Ischemia	101.7±1.8**	23.2±1.5	30.3±0.7**	5.9±0.4	16.5±0.6**	20.5**±** 1.0**	3.5**±** 0.2**
Ocular HPC+ischemia	118.2±4.6* ^,^ a	28.8±1.6	34.6±1.2**	6.8±0.3	20.3±0.4* ^,^ b	29.5**±** 1.5^a^	5.3**±**0.2 a

Effect of ocular HPC on total retinal and retinal layer thicknesses (in µm) in non-ischemic or ischemic eyes. Ischemia decreased total retinal, ONL, INL and IPL thickness, and GCL Brn3a(+) cell number. Ocular HPC which showed no effects in non-ischemic eyes, prevented the alterations in total retinal, INL, and IPL (but not ONL) thickness, and in retinal ganglion cell number. Data are mean ± SEM (n = 5 eyes per group).

* *P*<0.05 and,

** *P*<0.01 vs non-ischemic eyes;

a p<0.05 and

b p<0.01 vs. ischemic eyes without ocular HPC, by Tukey’s test.

PS, photoreceptor outer and inner segment; OPL, outer plexiform layer; ONL, outer nuclear layer; IPL, inner plexiform layer; INL, inner nuclear layer.

### Involvement of Glutamate in Ocular Hypothermic Preconditioning

**Figure 6:**
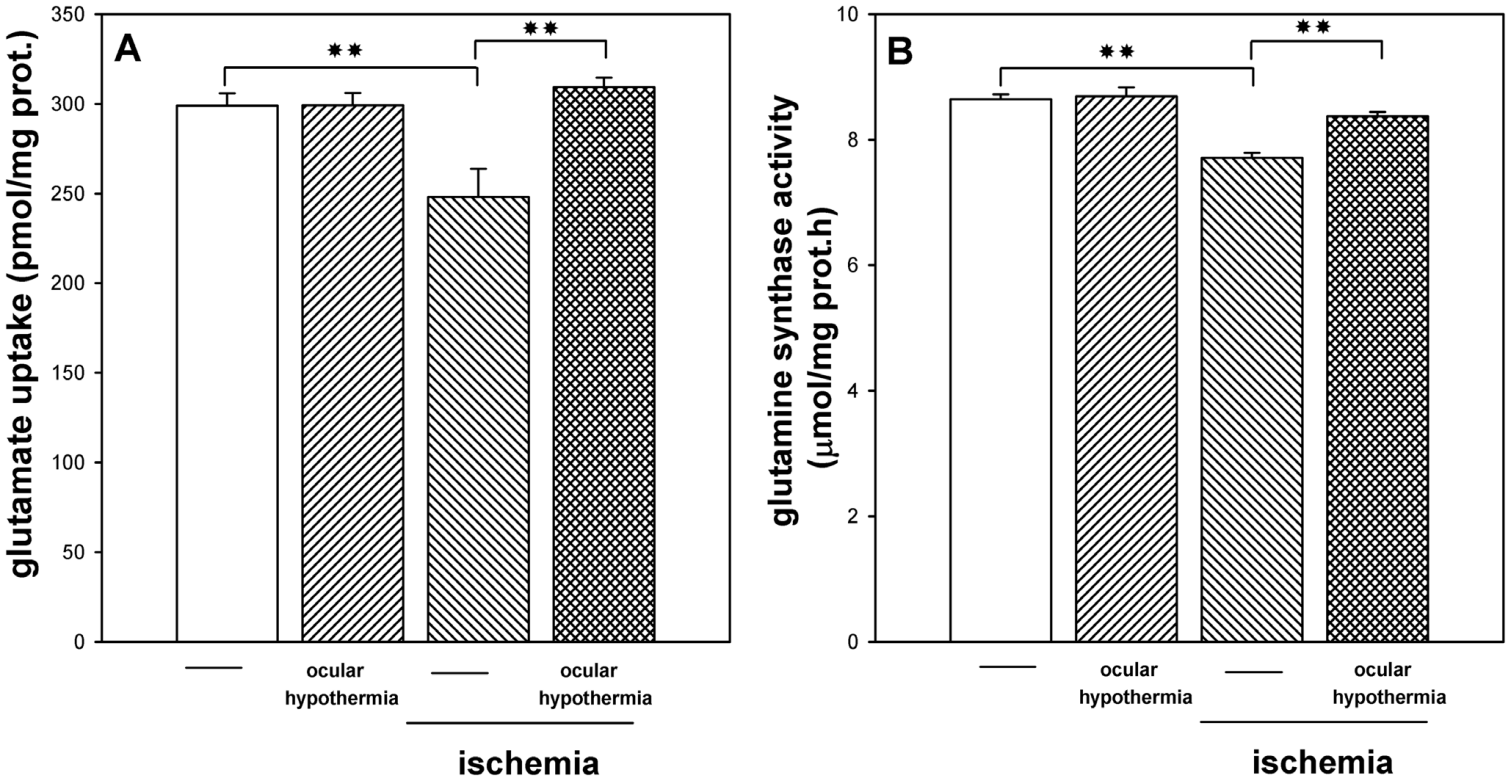
Effect of ischemia and ocular HPC on retinal glutamate uptake and glutamine synthetase activity. Three days after ischemia, a significant decrease of glutamate uptake (panel A) and glutamine synthetase activity (panel B) was observed in ischemic retinas, whereas local HPC, which showed no effect *per se* in non-ischemic eyes, significantly prevented the effect of ischemia on these parameters. Data are mean ± SEM (n = 10–12 animals per group). **P<0.01, by Tukey’s test.

Figure 6 shows L-^3^H-glutamate uptake and glutamine synthetase activity in retinas from control eyes and eyes submitted to ischemia without or with ocular hypothermia applied 24 h before ischemia, and assessed 3 days after ischemia. These parameters significantly decreased in ischemic retinas as compared with non-ischemic retinas. Ocular hypothermia, which did not affect these parameters in non-ischemic eyes, significantly prevented the effect of ischemia.

**Figure 7:**
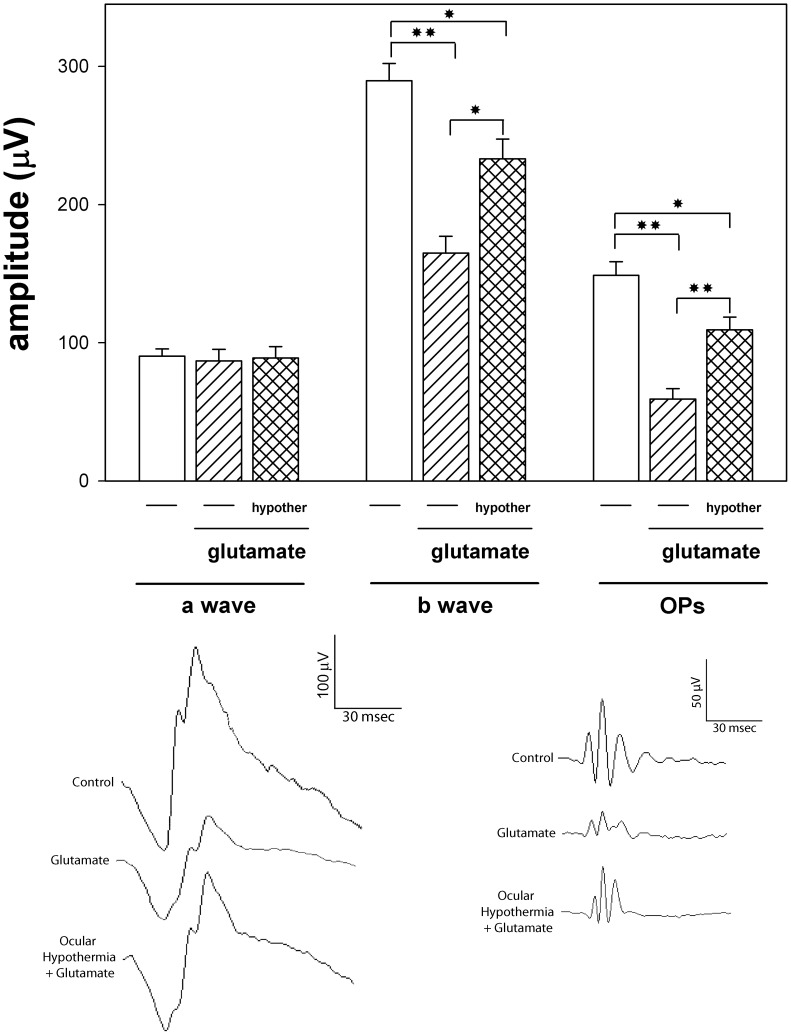
Effect of an intravitreal injection of glutamate without or with local HPC on retinal function. Eyes were intravitreally injected with vehicle or glutamate without or with local HPC applied 24 h before injections. Glutamate induced a significant decrease in the ERG b-wave and OP amplitude, whereas local HPC significantly prevented the effect of glutamate on these parameters. Upper panel: Average amplitudes of scotopic ERG a- and b-wave, and OP amplitudes. Shown are means ± SEM (n = 10 animals/group). *P<0.05, and **P<0.01, by Tukey’s test. Lower panel: Representative scotopic ERG and OP traces from eyes injected with glutamate without or with local HPC.

**Figure 8:**
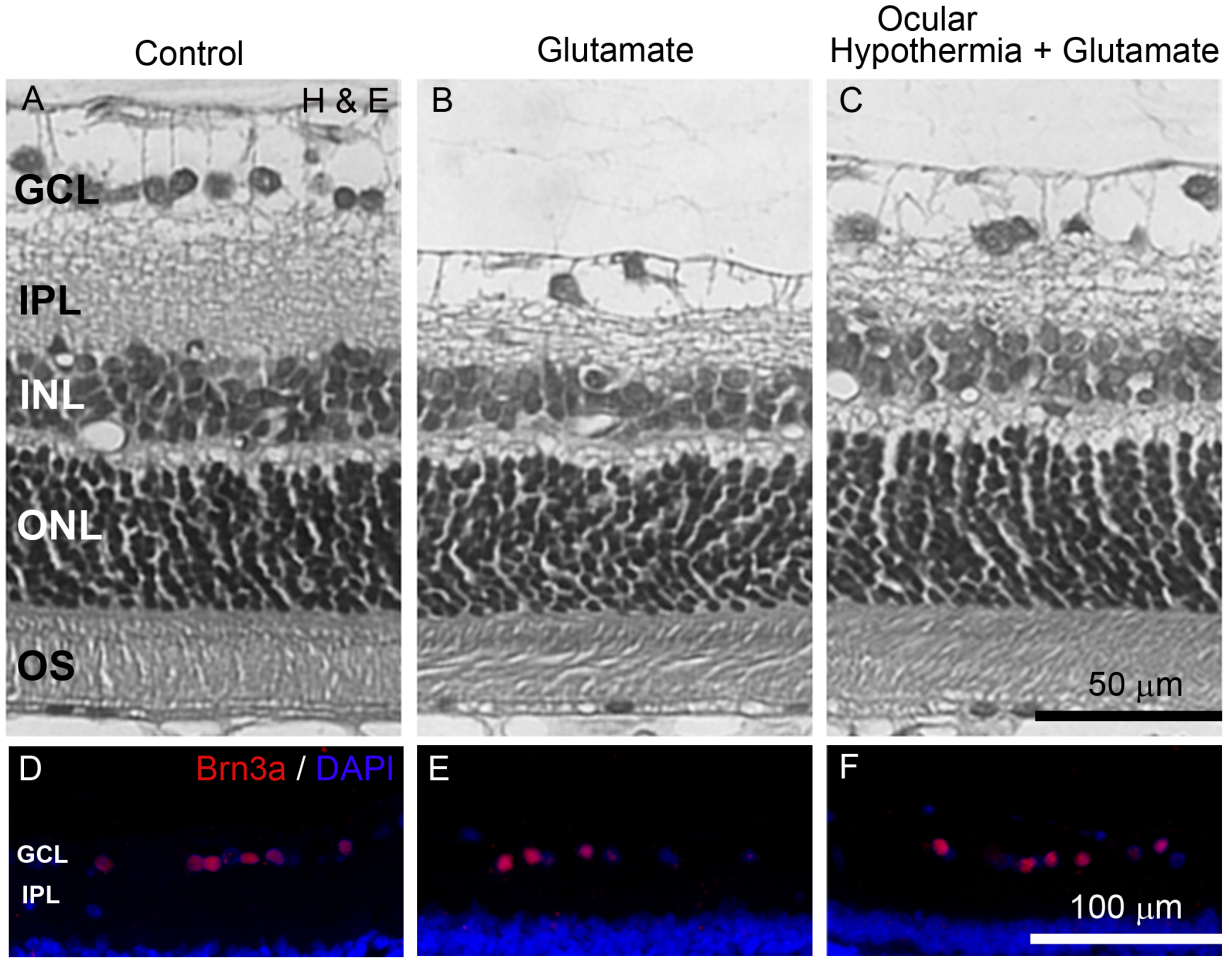
Effect of glutamate without or with local HPC on retinal histology. Representative photomicrographs showing histological appearance of retinas obtained 7 days after injection of vehicle (A, D) or glutamate without (B, E) or with local HPC applied 24 h before glutamate injection (C, F). Glutamate induced alteration in the retinal structure (upper panel) and GCL Brn3a(+) cell number (lower panel), whereas local HPC prevented histological alterations from glutamate damage. GCL, ganglion cell layer; IPL, inner plexiform layer; INL, inner nuclear layer; ONL, outer nuclear layer, OS, outer segment of photoreceptors.

The effect of an intravitreal injection of glutamate without or with ocular HPC on scotopic ERG and OPs is shown in Figure 7. Glutamate induced a significant decrease in the ERG b-wave and OP amplitude (but not their latencies or the a-wave amplitude) as compared with vehicle-injected eyes, whereas ocular hypothermia applied 24 h before glutamate injection significantly abrogated the effect of glutamate. The effect of an intravitreal injection of glutamate without or with ocular hypothermia applied 24 h before glutamate injection on retinal histology is depicted in Figure 8 and Table 3. Glutamate induced a significant decrease in the total retinal, INL, and IPL thickness, and in Brn3a(+) cell number (Figure 8B and 8E) as compared with vehicle injected eyes (Figure 8A and 8D), whereas ocular hypothermia prevented the effect of glutamate on these parameters (Figure 8C and 8F). Similar results in retinal function and histology were observed in non-injected eyes and eyes intravitreally injected with vehicle (data not shown).

**Table 3:** Protective effect of ocular HPC on retinal damage induced by glutamate.

	Total Thickness	PS	ONL	OPL	INL	IPL	Brn3a (+) cell number
Vehicle	133.9±3.5	27.5±1.1	41.6±0.5	6.4±0.3	20.5±0.4	35.3**±**0.5	5.2**±**0.2
Glutamate	119.4±2.0**	28.8±0.3	43.5±0.6	8.1±1.0	18.5±0.3**	19.4**±**0.8**	3.4**±**0.3**
Ocular HPC+Glutamate	131.7±2.8	30.4±1.7	43.3±0.6	8.8±0.2	21.9±0.3^b^	22.7**±** 0.7** ^,^ a	4.6**±** 0.3* ^,^ a

Total retinal and retinal layer thicknesses (in µm) and retinal ganglion cell number in vehicle- or glutamate-injected eyes without or with ocular HPC. Glutamate induced a decrease in total retinal, INL, and IPL thickness and Brn3a(+) cell number, which was significantly prevented by ocular HPC. Data are mean ± SEM (n = 5 eyes per group).

* *P*<0.05,

** *P* <0.01 vs. vehicle-injected eyes,

a *P* <0.05, and,

b *P*<0.01 vs. glutamate-injected eyes without ocular HPC, by Tukey’s test.

PS, photoreceptor outer and inner segment; OPL, outer plexiform layer; ONL, outer nuclear layer; IPL, inner plexiform layer; INL, inner nuclear layer; GCL, ganglion cell layer.

## Discussion

For the first time, the present results indicate that transient global or ocular hypothermia applied 24 h before an ischemic event, significantly prevented the effect of I/R on retinal function and histology. Brn3a is a POU domain transcription factor that is specifically expressed in the nuclei of RGCs [27]. In the retinas from ischemic eyes, a significant loss of RGCs was observed, as shown by Brn3a immunohistochemistry, whereas global or ocular HPC significantly prevented the loss of RGCs induced by I/R.

Hypothermia is one of the oldest, yet most effective, intraischemic treatments for limiting cellular injury. Systemic or focal cooling has been shown to provide a beneficial effect in the context of cerebrovascular, cardiovascular, and other types of surgeries [28]–[30]. In experimental studies, hypothermia has been referred to as “the gold standard” against which other ischemic therapies should be compared [31], [32] and has been described as “the most potent therapeutic approach for reducing experimental ischemic brain injury identified to date” [31]. These descriptions refer to the well established protective effects of hypothermia when delivered during ischemia in several systems, including the retina [20], [24]. The present results indicate that transient global or ocular hypothermia can also protect the retina when applied 24 h before an ischemic event, which indicate that the simultaneity of hypothermia with ischemia is not a necessary requirement for its protective effect. Preconditioning differs from neuroprotection in several aspects. The prophylactic approaches to neuroprotection in essence represent acute or chronic pretreatments in which the drug or manipulation are present when damage occurs, whereas in preconditioning, the singular or final treatment precedes the deleterious event by many hours or days, and the obligatory genomic reprogramming that largely defines the tolerant phenotype is promoted [13]. Thus, it seems likely that hypothermia could behave as a neuroprotectant or a preconditioning stimulus, depending on the time of its application with respect to the ischemic episode.

Several stimuli are able to induce retinal ischemic tolerance, such as LPS [11], inhaled carbon monoxide [33], and hyperbaric oxygen [34], among others. Unfortunately, most stimuli that induce tolerance are also capable of inflicting injury if their intensity or duration is slightly increased. It is therefore of considerable importance to identify effective preconditioning stimuli that have better safety margins and entail less risk. Although deep systemic hypothermia may have several side effects, including increased peripheral vascular resistance, increased cardiac afterload, bradycardia, acidosis, increased blood viscosity, cerebrovascular constriction, and abnormalities in blood coagulation [35], [36], these side effects are limited and/or manageable at mild-to-moderate levels of hypothermia [18], as those used in this report. In fact, the safety of even longer periods and of higher magnitude of hypothermia than those used herein has been shown in the brain from rodents and humans [17], [18], [37]–[39]. In this vein, it was shown that as many as 3 h of lowering the temperature at 30°C does not provoke damage to the brain [40]. In the experimental conditions used in this report, no significant effects of global or ocular hypothermia were observed in non-ischemic eyes at functional and histological level, supporting the safety of these procedures. Moreover, several clinical reports demonstrate that prolonged periods (that is, days-weeks) of hypothermia in a similar temperature range (32–33°C) are well tolerated by patients [41]–[43], which suggest that HPC could potentially serve as a tolerance-inducing stimulus in a clinical setting.

Yunoki et al. [18] showed a similar magnitude of protection against brain ischemia by focal or global cooling. However, at the experimental conditions used in this report, global HPC appeared to be more effective than ocular HPC in the protection of retinal function against ischemic damage. There is no ready explanation for this discrepancy. It has been demonstrated that repeated cycles of temporary ischemia in a remote organ (i.e. remote preconditioning) can activate protective pathways in the target organ, including the heart [44] and brain [45]. Therefore, although there are no data on the neuroprotective effect of remote preconditioning in the retina, it is tempting to speculate that besides ocular cooling, global HPC could protect the retina by a remote preconditioning mechanism. Notwithstanding, it seems likely that by changing the experimental conditions (time and temperature), it would be possible to achieve a higher magnitude of protection by ocular HPC which, contrary to hypothermia applied through whole body, is not associated with hemodynamic imbalance, and can be used to minimize side effects of systemic hypothermia, eventually, without significantly compromising the magnitude of the tolerance effect.

It has been demonstrated that retinas subjected to mild or moderate hypothermia had a decrement in neuronal energy metabolism which is fully restored once the retinas were placed under normothermic conditions [46]. However, since global or ocular HPC were applied 24 h before the ischemic episode, it seems unlikely that the reduction in retinal energy usage could account for the protective effect of HPC.

Preconditioning stimuli capable of eliciting tolerance are, for the most part, rather nonspecific in their actions on target tissues. It would clearly be of benefit to identify the mechanistic underpinnings of ischemic tolerance to develop more specific and clinically applicable therapies. The link between excitotoxicity and retinal ischemic damage had received considerable experimental support. Glial cells, mainly astrocytes and Müller cells, surround glutamatergic synapses, and express glutamate transporters and the glutamate-metabolizing enzyme, glutamine synthetase [46]–[48]. Glutamate is transported into glial cells and amidated by glutamine synthetase to the non-toxic aminoacid glutamine. Glutamine is then released by glial cells and taken up by neurons, where it is hydrolyzed by glutaminase to form glutamate again, completing the retinal glutamate/glutamine cycle [49], [50]. In this way, the neurotransmitter pool is replenished and glutamate neurotoxicity is prevented. In order to get insight into the mechanism involved in the protective effect of retinal HPC, glutamate uptake and glutamine synthetase activity were assessed in ischemic retinas without or with ocular HPC. As previously shown, retinal ischemia induced a significant decrease in both parameters [2] which was prevented by ocular HPC. In this way, the decrease in glutamate uptake and glutamine synthetase activity in ischemic retinas could contribute to an excessive increase in glutamate synaptic level, whereas the enhancement of glutamate influx and glutamine synthetase activity by ocular HPC may protect the retina from I/R injury. Moreover, as shown herein, the intravitreal injection of supraphysiological concentrations of glutamate provoked significant alterations in retinal function and histology which were prevented by ocular HPC. Thus, without excluding the activation of other transduction pathways involved in retinal ischemic tolerance, such as nitric oxide [11], adenosine [51], K_ATP_ channels [52], PKC [52], and vascular endothelial growth factor [53], among others, these results support that reducing glutamate synaptic levels could represent a crucial step in the retinal protection induced by HPC against ischemic damage. At retinal level, glutamate uptake and glutamine synthetase are mainly localized in Müller’s cells [54]. Therefore, the fact that HPC prevented the effect of I/R on glutamate uptake and glutamine synthetase activity, points at Müller cells as putative target for the protective effect of HPC on I/R damage, albeit the involvement of other cellular type(s) cannot be ruled out. Moreover, since ocular HPC was able to protect retinal function and histology against glutamate-induced neurotoxicity, HPC could have promise for application in retinal diseases which involve excitotoxicity-mediated retinal cell death.
